# Star Excursion Balance Test as a Predictor of Musculoskeletal Injury and Lower Back Pain in Non-Professional Soccer Players

**DOI:** 10.3390/sports11070129

**Published:** 2023-07-06

**Authors:** Iva Sklempe Kokic, Katarina Petric, Danijela Kuna, Stjepan Jelica, Tomislav Kokic

**Affiliations:** 1 Faculty of Kinesiology, Josip Juraj Strossmayer University of Osijek, 31000 Osijek, Croatia; iva.sklempe.kokic@kifos.hr (I.S.K.); dkuna@kifos.hr (D.K.); 2 Faculty of Health Studies, University of Rijeka, 51000 Rijeka, Croatia; katarina.petric14@gmail.com; 3 Department of Health Studies, College of Applied Sciences, “Lavoslav Ruzicka” in Vukovar, 32000 Vukovar, Croatia; sjelica@vevu.hr; 4 Faculty of Medicine, Josip Juraj Strossmayer University of Osijek, 31000 Osijek, Croatia; 5 Department of Orthopaedics and Traumatology, County General Hospital Vinkovci, 32100 Vinkovci, Croatia

**Keywords:** football, athletes, injuries, prevention, screening

## Abstract

Soccer is a sport with worldwide popularity but has a substantial risk of injury. Clinical screening tools are an important factor in strategies of injury prevention. The purpose of the study was to examine the relationship between the Star Excursion Balance Test (SEBT) and injury and lower back pain (LBP) in amateur soccer players. The research was performed as a longitudinal cohort study on 42 amateur male soccer players with 15.8 ± 6.6 years of soccer playing (age: 25.5 ± 6 years). Participants were surveyed with regard to their soccer playing, injuries, and LBP, and the SEBT was performed. They were followed for 3.5 months. At the follow-up, an additional set of data regarding injuries and LBP was gathered. Significant differences were found in all directions and in the composite score of the SEBT between uninjured and injured players. Shorter distance in all directions and a lower composite score were associated with injury in general. Shorter distances in all but the anterior direction and a lower composite score were associated with lower extremity injury, and shorter anterior distance was associated with LBP. Amateur soccer players with lower SEBT scores are more prone to injuries in general, as well as injuries of the lower extremities. SEBT presents as a useful clinical screening tool in identifying amateur soccer players at risk of injury.

## 1. Introduction

Soccer is a sport with worldwide popularity; however, the risk of injuries during practice and the game is high. The incidence of injuries in amateur soccer players ranges from 2.7 to 4.5 per 1000 h of practice and from 12.3 to 24.7 per 1000 h of game time [1,2,3]. The majority of soccer related injuries (68–88%) occur in the lower extremities [4,5]. Lower back pain (LBP) is another very common musculoskeletal disorder in soccer players with a yearly prevalence of 64% [6]. Participation in soccer can also have a significant role in the development of strength asymmetries leading to injury occurrence [7].

Prevention of injuries has an important role in reducing the injury burden in non-professional soccer players [8,9]. The use of clinical screening tools became an important component in the prevention of sports injuries. Previous studies have suggested that the use of an injury-screening tool related to dynamic balance may be beneficial in the identification of the risk of injury [10,11,12,13]. Dynamic balance can be defined as an individual’s ability to maintain total body stability of their center of mass during movement and is an integral part of neuromuscular control [14]. Deficits in lower extremity dynamic neuromuscular control, including impaired dynamic balance, is a known risk factor for injury [13,15,16]. Furthermore, dynamic balance is also impaired in individuals with LBP [17,18]. Dynamic neuromuscular control is a frequently utilized component of injury prevention programs as well as an outcome measure for return to sport criterion [10].

There is the need to further establish the relationship between dynamic balance and the risk of musculoskeletal injury and LBP in the amateur athlete population and to assess whether a clinical screening test for dynamic balance can be useful in the prediction of injuries and LBP. A valid and reliable screening tool for dynamic balance could be used in identifying those at risk and in the planning of preventive strategies. One of the very common screening tools for dynamic balance is the Star Excursion Balance Test (SEBT) [13,19]. The SEBT evaluates dynamic postural stability [20]. It is an inexpensive, simple, and quick method of measuring dynamic balance with good reliability reported [19,21]. In addition to dynamic balance, SEBT also requires other characteristics of neuromuscular control, such as coordination, flexibility, and strength, and each reach direction activates muscles in a different pattern [13,22].

Previous studies reported that reach asymmetries in anterior, posteromedial, and posterolateral direction of ≥4 cm, a normalized composite score <89.6%, and shorter individual reach distances during the SEBT and modified SEBT were associated with future injury risk [10,16]. However, the majority of the studies were not performed on non-professional athletes. Current information regarding the predictive value of SEBT for musculoskeletal injury and LBP in amateur athletes is still limited.

Therefore, the purpose of this study was to examine the relationship between SEBT reach distances and musculoskeletal injury and LBP among non-professional soccer players. We hypothesized that the SEBT can be used in the prediction of musculoskeletal injury and LBP in this population, and that there will be significant associations between shorter reach distances and injuries and LBP.

## 2. Materials and Methods

### 2.1. Study Design and Participants

The study was performed as a longitudinal cohort study on 42 amateur male soccer players from three amateur soccer clubs in Eastern Croatia. All three soccer clubs are part of the regional league, and they compete at the regional level. Ethical approval for the study was obtained from the Ethics Committee for Biomedical Research, Faculty of Health Studies, University of Rijeka, Croatia (21 February 2019). The study was carried out in accordance with the Declaration of Helsinki and participants gave their written consent. The inclusion criteria for participants were as follows: amateur soccer player, regular practice and games in the soccer club for at least six months before the beginning of the study, and an active soccer player for at least 5 years. Exclusion criteria were injuries or painful symptoms in the period of performing the screening tests and any other serious medical conditions, including surgery or concussion, six months before the testing. 

### 2.2. Procedures

Participants completed the questionnaire with the baseline information, which included demographic data, body height and body mass, health-related history, duration of soccer practice, training loads in the past six months, existence of injuries and lower back pain in the past six months, as well as leg dominance. Leg length was measured on each lower limb 3 times, from the greater trochanter to the lateral malleolus. Body mass index was obtained using the standard equation. After the initial interview, participants performed the SEBT after 10 min of warm up. Participants were followed up 3.5 months after the initial interview with another questionnaire, which included information on training loads, injuries, and lower back pain in the period after the initial interview. An injury was defined as any injury sustained during training or competition resulting in restricted performance or time lost from play [23]. Lower back pain (LBP) was defined as pain and discomfort localized below the costal margin and above the inferior gluteal folds, with or without leg pain [24].

### 2.3. Star Excursion Balance Test

The Star Excursion Balance Test comprises a single-leg balance with an oppositional reaching movement measuring the anterior, posterolateral, and posteromedial reach of both legs [13]. The test was explained and demonstrated to the participants. Before the formal testing, they practiced six trials in each direction. Participants were positioned with their foot in the center of the testing grid, which was created by aligning a series of three tape measures secured on the floor. Athletes were instructed to keep their hands on their hips, head facing forward, and to keep their stance foot flat on the floor. In that position they were instructed to reach as far as possible in the three directions with the toe of the other foot and make a single, light toe touch on the tape measure. 

Reach distance was marked by the tape with the pen and then measured using a tape measure. The maximum value of three trials was used for analysis. The trial was repeated if the athlete failed to maintain a unilateral stance, lifted or moved the stance foot from the center of the grid, touched down with the reach foot, or failed to return the reach foot to the starting position. The distance reached was normalized to leg length by dividing excursion distance by leg length and they multiplying it by 100. The sum of the three reach directions divided by three times the leg length and then multiplied by 100 was used to calculate the composite scores.

### 2.4. Statistical Analysis

Statistical analysis was performed using SPSS 25.0 (IBM, Armonk, NY, USA). The normality of the data was checked using Shapiro–Wilk test. Descriptive statistics included the mean and standard deviation for numeric variables or frequency and percentages for categorical variables. 

The Welch test was used for comparison of the SEBT scores between the group of participants without injury and the group of participants with injury in general or specific injuries and LBP. Differences in the injury rate before and after the testing were calculated using McNemar’s test.

Binary logistic regression was initially performed to examine whether normalized SEBT scores for anterior, posteromedial, and posterolateral directions of both legs; composite scores and asymmetries ≥4 cm could identify those at risk of injury in general, lower extremity injury, contact lower leg injury, non-contact lower leg injury, and lower back pain. Odds ratio (OR) and 95% confidence intervals (CI) were calculated. 

Hierarchical multiple linear regression analyses were conducted to determine the impact of SEBT variables on injuries. Variables with a *p* value of <0.20 on the Wald test [25] in the univariate models were entered into multiple regression analyses. In these models age, previous injury, BMI, total training load, and number of training sessions per week were entered in Step 1, and SEBT variables were entered in Step 2. Tolerance and variance inflation factor (VIF) were used to check for multicollinearity. Results were considered significant at *p* < 0.05.

## 3. Results

Sixty amateur soccer players were invited to participate in the study. Forty-two players accepted the invitation and fulfilled the inclusion criteria. Table 1 shows the participant’s general characteristics. Regarding their tactical position in soccer, the majority of participants (N = 19; 45.2%) had the position of midfielder. Furthermore, there were 10 (23.8%) strikers, 7 (16.7%) defenders, and 6 (14.3%) goalkeepers. Their injuries and LBP before and after the testing are presented in Table 2. There were no significant differences in the injury and LBP rate between the period 6 months before the testing and at the end of the period 3.5 months after the testing.

The SEBT results for the total sample and according to injury status and LBP are presented in Table 3. Significant differences were found in all directions as well as in the composite score in the SEBT results between uninjured and injured players with any injury and lower extremity injury. In addition, significant differences were found in the anterior direction of both the dominant and non-dominant leg between those with and without LBP. None of the participants had anterior asymmetry ≥4 cm; however, 5 (11.9%) participants had posteromedial asymmetry ≥4 cm. Four (9.5%) participants had posterolateral asymmetry ≥4 cm. All but one participant had their SEBT score <89.6%.

Table 4 shows the SEBT results according to lower extremity injury mechanism. There were no significant differences between those with and without a contact and non-contact mechanism of injury.

Table 5 presents results of univariate logistic regression analysis for injuries and LBP as well as ORs with their 95% CI. Shorter distance in all directions of SEBT as well as a lower composite score for both the dominant and non-dominant lower extremities were associated with any injury. Shorter distance in all but the anterior direction of the dominant leg as well as lower composite scores were associated with lower extremity injury. Only the anterior reach of the non-dominant leg was associated with LBP.

The results of the univariate logistic regression analysis for contact and non-contact lower extremity injuries are shown in Table 6. No directions of the SEBT nor the composite score could correctly identify those at risk of contact or non-contact lower extremity injury. Posteromedial and posterolateral asymmetries ≥4 cm could not identify those at risk of injury or LPB.

Multiple regression analysis revealed that age, previous injury, BMI, total training load, and number of training sessions per week explained 28% of the injury variance, while the composite score for the non-dominant leg added an extra 13% (Table 7). There was a positive association between BMI and injury, and negative associations between training load, SEBT composite score of the non-dominant leg, and injury in general. Age, previous, injury, BMI, total training load, and number of training sessions per week explained 47% of the lower extremity injury variance. The composite score for the non-dominant leg added an extra of 11%. There were negative associations between training load and the SEBT composite score of the non-dominant leg and lower extremity injury. Other SEBT variables did not contribute to neither model.

## 4. Discussion

This study aimed to examine if there is a relationship between SEBT reach distances and musculoskeletal injury and LBP among non-professional soccer players. Furthermore, it aimed to determine whether the SEBT scores can be used in the prediction of injuries and LBP. The results partially confirmed our hypothesis. Shorter reach distances in all directions and lower composite score were associated with musculoskeletal injury in general. Likewise, shorter reach distances in all but the anterior direction of the dominant leg and a lower composite score were associated with lower extremity injury. LBP was associated with a shorter reach distance in the anterior direction of the non-dominant leg. However, SEBT results could not identify those at risk of specific lower extremity injuries. Asymmetries in posterolateral and posteromedial directions ≥4 cm could not identify those at risk of injury or LBP. To the best of our knowledge, only one previous study investigated predictive value of the SEBT in non-professional soccer players. This adds some new aspects regarding the association between the SEBT and musculoskeletal injury and LBP in very specific population, amateur soccer players, with the possibility to lower the risk of injuries and LBP by using screening and implementing prevention strategies according to the results of the screening. We specifically selected this population because there were not enough previous studies which included amateur athletes and data in the literature for professional athletes cannot be generalized to amateur athletes.

A previous similar study performed by Gonell et al. [26], which examined the relationship between modified SEBT scores and soft tissue injury incidence in a soccer team, included a total of 74 soccer players. Forty of these participants were amateur athletes. All reach directions, including the composite score, exceeded values of the results in our sample; however, the results were similar to ours, showing an increased risk of injury with decreased reach directions of the SEBT. A significant relationship was found between below the average normalized anterior reach distance and contact injuries, as well as below the average normalized posteromedial reach distance and non-contact injuries. Below the average values of the normalized posterolateral reach distance and normalized composite reach distance were related to an increased risk of total and non-contact injuries. Participants with a composite reach distance below the average of the sample were approximately two times more likely to sustain an injury. 

Another previous study examining the predictive value of SEBT scores on lower extremity injuries was performed by Plisky et al. [13]. They investigated whether SEBT reach distance was associated with the risk of lower extremity injury among high school basketball players, which makes their sample quite different than ours. However, their results were similar. They reported that an anterior right/left reach distance difference ≥4 cm, decreased the normalized right anterior reach distance and decreased the normalized posteromedial, posterolateral, and composite reach distances bilaterally were significantly associated with lower extremity injury. 

O’Connor et al. [27] examined whether modified SEBT results can identify those at risk of contact or non-contact lower extremity injury in a population of adolescent and collegiate footballers and hurlers. Poor scores were unable to ascertain those at risk of contact or non-contact lower extremity combined and ankle injuries with sufficient sensitivity. Contrary to that, a study performed by Ko et al. [28] established that the posteromedial and posterolateral reach of the SEBT can identify adolescent athletes who will incur a subsequent lateral ankle sprain. Indeed, the literature reported that asymmetry in the anterior direction is more common among those with a history of lateral ankle sprain in comparison to those without [29]. In our study, we did not find asymmetries related to future injuries; however, ankle sprain was not specifically taken into account.

Research by Ganesh et al. [18] reported on significant reductions in excursion distances for all directions of the SEBT in group of subjects with lower back pain. Contrary to that, another study investigating SEBT results in young athletes with back pain did not find significant differences between the reach distances in all three directions of those with and without back pain [30]. In our study, we find only differences in the anterior reach for both the dominant and non-dominant leg between those with and without LBP. In addition, a shorter distance of the anterior reach of the non-dominant leg was associated with the occurrence of LBP.

Indeed, impaired balance is established as a risk factor for lower limb injury [31]. Neuromuscular risk factors for lower extremity injury in male soccer players include m. quadriceps dominance, leg dominance (asymmetry), knee valgus, trunk dominance, and reduced dynamic stability [32]. Furthermore, previous research reported impaired postural control in LBP and proposed that balance impairment in LBP patients correlates with deficits in the musculoskeletal and neural systems [33,34]. Controlling balance requires the interaction of the neurological, musculoskeletal, proprioceptive, vestibular, and visual systems [18]. Therefore, it is very important to develop and validate appropriate screening methods for assessments of neuromuscular control, including balance, with the goal of identifying those which may be at a greater risk of injury or LBP.

We have demonstrated that the SEBT can be a useful and simple method of clinical screening in amateur soccer players. The test could be incorporated into the pre-participation screening of athletes to help identify those at risk of injury or LBP. These deficits could be improved through a neuromuscular training program prior to the competitive season. There is the need to establish cut-off points, which will be population- and sport-specific, in order to more accurately determine the future injury risk. Cut-off points and previous research on professional soccer players are not generalizable to the amateur athlete population due to different training loads and the frequency of training sessions and competitions.

Our study’s prospective design allowed us to minimize recall bias regarding the injuries. However, our study had some limitations. First, our sample was small and consisted of only male amateur soccer players from three soccer clubs. This makes difficult to generalize our results to other populations of athletes. Furthermore, the follow-up period was relatively short. Future studies should consider a larger sample consisting of both genders from different sports, and a longer period of follow up. It is also important to note that we examined only the results of the SEBT in terms of injury prediction, and we did not take into account many of the other variables that could influence the risk of injury and LBP. Strong points of the study include the use of a valid and reliable test, a homogenous population, and the prospective design.

## 5. Conclusions

In conclusion, our data suggests the predictive value of the SEBT for injuries in general, lower extremity injuries, and LBP occurrence. It seems that amateur soccer players with lower SEBT scores are more prone to injuries in general, as well as injuries of the lower extremities. Amateur soccer players have different training loads and lifestyle in comparison to professional athletes, and there is also lack of financial resources for the amateur soccer clubs. However, use of simple screening procedures could have a positive impact on injuries and LBP. Further studies are needed to confirm these findings and to establish specific cut-off points, which would allow more precise predictions.

## Figures and Tables

**Table 1:** General characteristics of the participants (N = 42).

Variable	Mean ± SD
Age (years)	25.5 ± 6
Body height (cm)	181 ± 6.3
Body mass (kg)	78.8 ± 8.1
BMI (kg/m^2^)	24 ± 2.1
Duration of soccer playing (years)	15.8 ± 6.6
Weekly training load six months before screening tests (min)	334.5 ± 134.6
Weekly training load three months after screening tests (min)	310.5 ± 131.7

N—sample; SD—standard deviation; BMI—body mass index.

**Table 2:** Characteristics of the injuries and lower back pain among the participants (N = 42).

Variable	Period 6 Months before Testing (N (%))	Period 3.5 Months after Testing (N (%))	*p*
Injury of any type	14 (33.3)	14 (33.3)	1.000
LE injury	11 (26.2)	11 (26.2)	1.000
Contact LE injury	2 (4.8)	5 (11.9)	0.375
Non-contact LE injury	7 (16.7)	6 (14.3)	1.000
LBP	6 (14.3)	5 (11.9)	1.000

N—sample; LE—lower extremity; LBP—lower back pain.

**Table 3:** SEBT results for the total sample and according to injury status and LBP.

SEBT Results	Total Sample (N = 42)	No Injury (N = 28)	Any Injury (N = 14)	*p*	No LE Injury (N = 31)	LE Injury (N = 11)	*p*	No LBP (N = 37)	LBP (N = 5)	*p*
Dominant LE (LL (%))	mean ± SD	mean ± SD	mean ± SD		mean ± SD	mean ± SD		mean ± SD	mean ± SD	
ANT	58.5 ± 2.5	59.1 ± 2.3	57.4 ± 2.6	0.050 *	59 ± 2.3	57.2 ± 2.7	0.073 *	58.8 ± 2.5	56.4 ± 1.4	0.013 *
PMED	99.2 ± 4.5	100.7 ± 3.8	96.2 ± 4.6	0.005 *	100.3 ± 4.1	96.2 ± 4.5	0.018 *	99.6 ± 4.5	96.1 ± 4.2	0.142
PLAT	96.2 ± 3.8	97.3 ± 3.1	94 ± 4.2	0.017 *	97.1 ± 3.4	93.9 ± 3.9	0.032 *	96.6 ± 3.6	93.6 ± 4.7	0.227
Composite	84.7 ± 3.1	85.7 ± 2.2	82.6 ± 3.6	0.008 *	85.4 ± 2.6	82.5 ± 3.4	0.021 *	85 ± 3	82 ± 3.3	0.115
Non-Dominant LE (LL (%))	mean ± SD	mean ± SD	mean ± SD		mean ± SD	mean ± SD		mean ± SD	mean ± SD	
ANT	59.4 ± 2.5	60.1 ± 2.3	58.1 ± 2.5	0.019 *	60 ± 2.5	57.9 ± 2.2	0.020 *	59.8 ± 2.5	56.9 ± 1.3	0.003 *
PMED	100.4 ± 4.2	101.7 ± 3.4	97.7 ± 4.3	0.006 *	101.4 ± 3.8	97.4 ± 3.8	0.007 *	100.7 ± 4.1	98 ± 4.4	0.247
PLAT	97.2 ± 3.7	98.3 ± 3.1	95 ± 3.7	0.008 *	98 ± 3.4	94.9 ± 3.4	0.019 *	97.5 ± 3.5	95 ± 4.6	0.299
Composite	85.7 ± 2.9	85.7 ± 2.2	83.6 ± 3.3	0.004 *	86.5 ± 2.5	83.4 ± 2.8	0.006 *	86 ± 2.8	83.3 ± 3.2	0.131

N—sample; SD—standard deviation; LE—lower extremity; LBP—low back pain; ANT—anterior; PMED—posteromedial; PLAT—posterolateral; LL—normalized score; * statistically significant.

**Table 4:** SEBT results according to LE injury mechanism.

SEBT Results	No LE Contact Injury (N = 37)	LE Contact Injury (N = 5)	*p*	No LE Non-Contact Injury (N = 36)	LE Non-Contact Injury (N = 6)	*p*
Dominant LE (LL (%))	mean ± SD	mean ± SD		mean ± SD	mean ± SD	
ANT	58.8 ± 2.4	56.7 ± 2.8	0.172	58.7 ± 2.5	57.7 ± 2.7	0.450
PMED	99.6 ± 4.5	96.2 ± 3.8	0.118	100 ± 4.3	96.3 ± 5.3	0.183
PLAT	96.6 ± 3.8	93.9 ± 3.6	0.184	96.6 ± 3.6	93.9 ± 4.5	0.213
Composite	85 ± 3	82.3 ± 3.1	0.120	85 ± 2.9	82.7 ± 4	0.219
Non-Dominant LE (LL (%))	mean ± SD	mean ± SD		mean ± SD	mean ± SD	
ANT	59.6 ± 2.5	57.9 ± 2.3	0.166	59.7 ± 2.3	58 ± 2.3	0.152
PMED	100.8 ± 4	96.8 ± 3.7	0.074	100.8 ± 4	97.8 ± 4.2	0.152
PLAT	97.5 ± 3.7	95 ± 2.9	0.121	97.6 ± 3.5	94.9 ± 4.1	0.179
Composite	86 ± 2.8	83.2 ± 2.8	0.088	86 ± 2.8	83.6 ± 3.1	0.113

N—sample; SD—standard deviation; LE—lower extremity; ANT—anterior; PMED—posteromedial; PLAT—posterolateral; LL—normalized score.

**Table 5:** Univariate logistic regression analysis for injuries and LBP.

SEBT Results	Any Injury			LE Injury			LBP		
	*p*	OR	95% CI	*p*	OR	95% CI	*p*	OR	95% CI
Dominant LE (LL (%))									
ANT	0.045 *	1.366	1.008–1.853	0.055	1.372	0.993–1.895	0.058	1.563	0.984–2.481
PMED	0.006 *	1.315	1.080–1.602	0.017 *	1.254	1.041–1.510	0.112	1.194	0.959–1.487
PLAT	0.013 *	1.305	1.057–1.612	0.026 *	1.279	1.030–1588	0.108	1.253	0.952–1.649
Composite	0.005 *	1.485	1.124–1.962	0.012 *	1.417	1.079–1.860	0.060	1.379	0.986–1.930
Non-Dominant LE (LL (%))									
ANT	0.022 *	1.470	1.056–2.046	0.031 *	1.468	1.036–2.082	0.028 *	1.886	1.071–3.319
PMED	0.007 *	1.307	1.077–1.587	0.010 *	1.294	1.062–1.577	0.183	1.164	0.931–1.455
PLAT	0.011 *	1.337	1.070–1.672	0.024 *	1.288	1.035–1.602	0.159	1.206	0.929–1.564
Composite	0.004 *	1.558	1.155–2.103	0.007 *	1.490	1.113–1.995	0.069	1.349	0.976–1.863

LE—lower extremity; LBP—low back pain; ANT—anterior; PMED—posteromedial; PLAT—posterolateral; LL—normalized score; * statistically significant.

**Table 6:** Univariate logistic regression analysis for contact and non-contact LE injuries.

SEBT Results	Contact LE Injury			Non-Contact LE Injury		
	*p*	OR	95% CI	*p*	OR	95% CI
Dominant LE (LL (%))						
ANT	0.089	1.466	0.944–2.279	0.388	1.173	0.816–1.685
PMED	0.119	1.119	0.956–1.480	0.095	1.190	0.970–1.461
PLAT	0.155	1.213	0.930–1.582	0.118	1.220	0.951–1.565
Composite	0.081	1.337	0.964–1.853	0.100	1.281	0.954–1.722
Non-Dominant LE (LL (%))						
ANT	0.147	1.380	0.893–2.133	0.147	1.342	0.902–1.998
PMED	0.060	1.259	0.991–1.601	0.115	1.185	0.960–1.464
PLAT	0.152	1.210	0.932–1.570	0.107	1.224	0.957–1.565
Composite	0.062	1.364	0.984–1.889	0.072	1.315	0.976–1.773

LE—lower extremity; ANT—anterior; PMED—posteromedial; PLAT—posterolateral; LL—normalized score.

**Table 7:** Multiple regression analysis examining the contribution of age, previous injury, BMI, training load, and selected SEBT variables to injury and LE injury.

Dependent Variable: Any Injury				Dependent Variable: LE Injury			
	b	SE b	β		b	SE b	β
Step 1				Step 1			
Constant	−1.926	0.855		Constant	−1.129	0.827	
Age	−0.006	0.013	−0.079	Age	−0.007	0.013	−0.088
Previous injury	0.302	0.152	0.302	Previous injury	0.291	0.147	0.312
BMI	0.085	0.035	0.378 *	BMI	0.050	0.033	0.240
Training load	−0.002	0.001	−0.685 *	Training load	−0.002	0.001	−0.712 *
Number of training sessions per week	0.281	0.162	0.515	Number of training sessions per week	0.272	0.157	0.534
Step 2				Step 2			
Constant	4.537	2.432		Constant	4.420	2.411	
Age	−0.012	0.012	−0.154	Age	−0.012	0.012	−0.164
Previous injury	0.106	0.155	0.106	Previous injury	0.123	0.154	0.132
BMI	0.073	0.032	0.323 *	BMI	0.040	0.032	0.189
Training load	−0.002	0.001	−0.583 *	Training load	−0.002	0.001	−0.618 *
Number of training sessions per week	0.278	0.149	0.510	Number of training sessions per week	0.270	0.147	0.530
Composite score of non-dominant leg	−0.070	0.025	−0.430 **	Composite score of non-dominant leg	−0.060	0.025	−0.396 *
R^2^ = 0.28 for Step 1: ΔR^2^ = 0.13 for Step 2 (*p* < 0.05)				R^2^ = 0.47 for Step 1: ΔR^2^ = 0.11 for Step 2 (*p* < 0.05)			

* *p* < 0.05, ** *p* < 0.01; SE—standard error; b—unstandardized coefficient; β—standardized coefficient.

## Data Availability

The data presented in this study are available on request from the corresponding author.
